# An unusual localization of retroperitoneal paraganglioma: a case report

**DOI:** 10.11604/pamj.2015.22.12.7437

**Published:** 2015-09-08

**Authors:** Mohamed Said Belhamidi, Moulay Brahim Ratbi, Mohamed Tarchouli, Tariq Adioui, Abdelmounaim Ait Ali, Aziz Zentar, Khalid Sair

**Affiliations:** 1 Department of General Surgery, Mohamed V Military Teaching Hospital, Mohamed V-Souissi University, Rabat, Morocco

**Keywords:** Retroperitoneal, paraganglioma, surgery

## Abstract

Paragangliomas are rare tumors arising from extra-adrenal chromaffine tissues. The diagnosis of non-functional retroperitoneal paraganglioma and its surgical management can be difficult. We report a case of a retroperitoneal paragangliomaof an unusual localization that renders the surgery more challenging. A 40 year-old woman presented to our department with a four-month history of upper quadrant pain with no vomiting, no fever, nor jaundice. Physical examination was normal. Ultrasonography showed a retro duodenal homogenous mass and computed tomographyscan showed a well-circumscribed round mass of heterogeneous density, which was in close contact with the aorta and the left kidney vein. Laboratory tests were normal. The patient underwent surgical management. The surgical exploration found a retroperitoneal tumor that was encapsulated and showing intimate contact with the abdominal aorta. We performed a complete resection of the tumor. Histological examination of the surgical specimen revealed a paraganglioma. The post operative course was uneventful. Paragangliomas are rare tumors. They can be asymptomatic for a long time and thus be diagnosed at late stage. A follow-up of patients is then essential. Surgical treatment is the only radical treatment and should be performed even in paragangliomas in close contact with the great vessels.

## Introduction

Paragangliomas are rare tumors arising from extra-adrenal chromaffine tissues, that is the paraganglia, which are **widely** distributed near or within the autonomic nervous system in a variety of retroperitoneal sites and in the sympathetic ganglia of various viscera. All paragangliomas are **believed** to be derivedfrom the neural crest; they can synthesize and store catecholamines. The diagnosis of non-functional retroperitoneal paraganglioma and its surgical management can be difficult. We report a case of a retroperitoneal paraganglioma of an unusual localization that renders the surgery more challenging.

## Patient and observation

**Figure 1:**
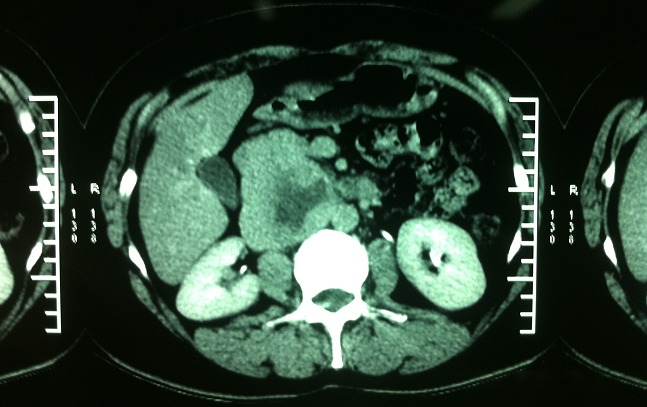
Axial CT scan image showing a round mass with a necrotic center, touching the aorta and inferior vena cava

**Figure 2:**
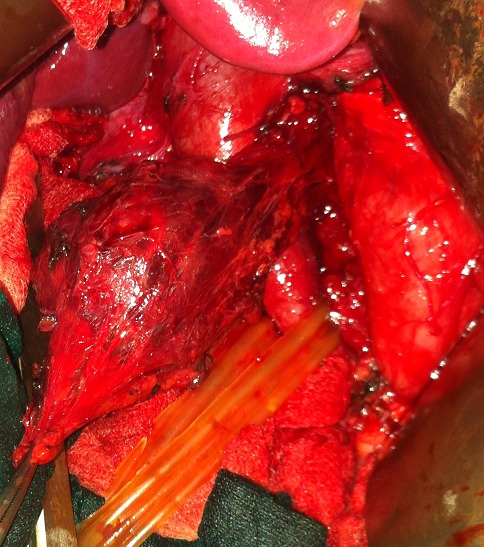
Tumor located in the inter aorticocaval area

**Figure 3:**
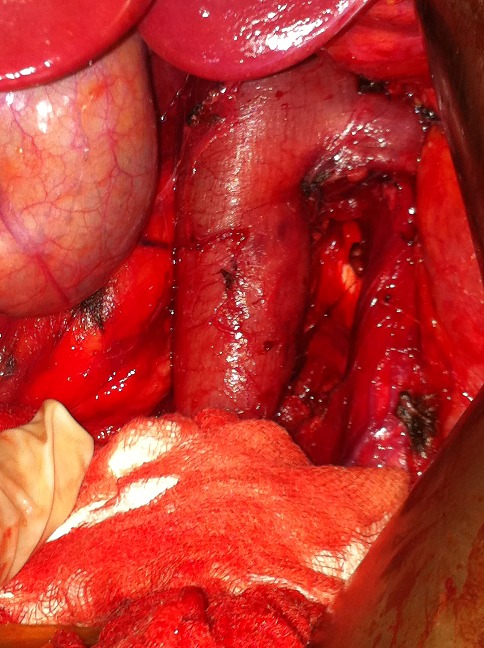
Peroperative view after tumor excision

**Figure 4:**
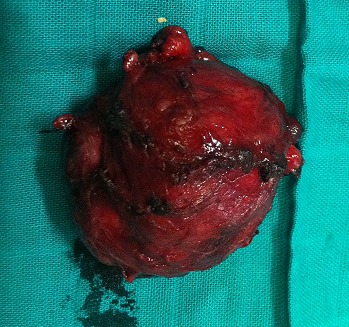
The excised tumor

A 40 year-old woman presented with a four-month history of upper quadrant pain with no vomitingnor jaundice. On examination, the patient had slight tenderness in the upper right quadrant with no palpable mass. She was afebrile with no pallor, no pedal edema nor peripheral lymphadenopathy. Ultrasonography showed a retro duodenal homogenous round mass, with no intra hepatic or common bile ducts dilatation. Abdominal computed tomography scan showed a well-circumscribed round mass of heterogeneous density that was in contact with the aorta and the left kidney vein (Figure 1). Laboratory tests showed leukocytosis and elevated C-reactive protein. Carcinoembryonic antigen and CA 19-9 were within normal range, methoxyl derivatives test were negative. The patient then underwent surgical management by a right subcostal approach. Per-operative exploration found a retroperitoneal tumor of about 8 cm in diameter that was soft and encapsulated. The tumor was in intimate contact with the abdominal aorta, the left renal vein, and the inferior vena cava (Figure 2). Aplane cleavage was found, that enabled us to performa complete resection of the tumor (Figure 3, Figure 4). Histological examination of the surgical specimen revealed a paraganglioma. The postoperative course was uneventful. The patient continued to be followed-up, regular physical and morphological examination (UltrasonographyAbdominal, computed tomography scan) did not find loco-regional recurrence or distant metastasis.

## Discussion

Paragangliomas are extra adrenal tumors composed of chromaffin cells that can secrete catecholamines. Retroperitoneal paragangliomas are a rare entity. It can occur at any age, most commonly in young adult [1, 2]. The functional symptoms are related to the secretion of catecholamines[3]. The main symptom is high blood pressure. The set of three, headache, palpitations, abounding sweats, is found in about 90 percent [4, 5]. Other symptoms are less suggestive: abdominal pain, anxiety, shivers, pallor and digestive disorder [4]. Our patient presented with an atypical upper quadrant pain without any specific symptoms. The preoperative diagnosis of the paraganglioma is essentiallybiological. The most sensitive and the most specific biological tests are the urinary dosage of metanephrine and normetanephrine and the dosage of the plasma free metanephrines. The dosage of chromogranin A is short specificity and sensitivity but can be useful in case of non-functional tumor and for the follow-up of the patients [6]. For extra adrenal sites, the dosage of metanephrine is often normal even for secreting tumors. The converting enzyme of normetanephrine andmetanephrine (phenylethanolamine -N- methyltransferase) is only active in adrenal tumors. In our patient, these tests were normal. The CT scan is an equivalent examination compared to the MRI in locating the tumor but is less competitive in the differential diagnosis with other tumors [3]. CT scans can show a retroperitoneal and retro duodenal mass typically solid, round and homogenous with, sometimes, necrotic or cystic areas in the center of tumor [7]. This is almost the same case with our patient in whom, CT showed a round pre aortic retroperitoneal tumor with a necrotic center.

Management of paragangliomas requires a multidisciplinary approach. Surgery is the only curative treatment provided that it is complete [8–10]. It allows survival rates of 75% and 45% at 5 and 10 years respectively [9]. Because of the vascularity of these tumors, some authors recommend the preoperative embolization of the tumor [11]. In our patient, although the tumor was in close contact with the aorta and inferior vena cava, the resection was complete. Other authors prefer laparoscopic resection of paragangliomas with all the known advantages of minimally invasive surgery [12]. Laparoscopy is only possible if the topography and vascular relationship of the tumor allows it. Laparoscopic management was not possible in our patient given the vascular relationship with the aorta and vena cava. Another problem is the characterization of the aggressiveness of paragangliomas. To determine a benign or malignant tumor, histological study is unreliable [9, 11, 13]. So far, for some authors, there were no histological criteria of malignancy and it was the appearance of distant metastases during follow-up of patient, which confirmed a malignancy of paraganglioma [9, 11]. In our patient, CT scan done six months after resection of the tumor shows no local recurrence or distant metastases. Despite the study of pleomorphism, mitotic activity or vascular invasion of the tumor, the evolution is unpredictable. Immunohistochemical studies for aggressiveness of these tumors are in progress. The prognosis may be dependent on the importance of the expression of some neuropeptides (Ki-67, Bax, and Bcl-2 and protein S-100) [13].

## Conclusion

Paragangliomas are rare tumors, most often observed in young adults. They can be asymptomatic for a long time and thus be diagnosed at late stage. Histology is often non-contributory to determining the benign or malignant nature of the tumor. The follow-up of patients is then necessary. Surgical treatment is the only radical treatment and should be performed even in paragangliomas in close contact with the great vessels.
